# Serum copper levels and risk of major adverse cardiovascular events: a systematic review and meta-analysis

**DOI:** 10.3389/fcvm.2023.1217748

**Published:** 2023-06-27

**Authors:** Carlos Muñoz-Bravo, Eva Soler-Iborte, Macarena Lozano-Lorca, Malak Kouiti, Carla González-Palacios Torres, Rocío Barrios-Rodríguez, José Juan Jiménez-Moleón

**Affiliations:** ^1^ Department of Public Health and Psychiatry, School of Medicine, University of Málaga, Málaga, Spain; ^2^ Biomedical Research Institute of Malaga (IBIMA), Málaga, Spain; ^3^ Department of Public Health, Hospital Universitario San Cecilio, Spain Biohealth Research Institute in Granada (ibs.GRANADA), Granada, Spain; ^4^ Department of Preventive Medicine and Public Health, Universidad de Granada, Granada, Spain; ^5^ Instituto de Investigación Biosanitaria Ibs.GRANADA, Granada, Spain; ^6^ Laboratory of Health Sciences and Technologies, Higher Institute of Health Sciences, Hassan First University of Settat, Settat, Morocco; ^7^ Consortium for Biomedical Research in Epidemiology and Public Health (CIBERESP), Madrid, Spain

**Keywords:** serum copper, cardiovascular disease, cardiovascular mortality, stroke, myocardial infarction, meta-analysis, major adverse cardiovascular events

## Abstract

**Background:**

Despite the fact that several studies have investigated the association between serum copper levels (S-Cu) and the risk of cardiovascular diseases, this relationship remains unclear. The aims of this study were to investigate the association between S-Cu and risk of major adverse cardiovascular events (MACE), including total stroke, ischemic stroke, hemorrhagic stroke, myocardial infarction and cardiovascular mortality, and identify potential sources of results heterogeneity.

**Methods:**

We carried out a systematic review and meta-analysis. The selection criteria were: (1) Observational studies (cohort studies, case-control studies and hybrid studies); (2) Studies containing quantitative data about the relationship between S-Cu and risk of MACE; (3) Estimating association measures; and (4) Studies written in English, French or Spanish. Overall pooled Odds ratio (pOR) and 95% confidence intervals (95% CI) of MACE for the highest vs. lowest S-Cu category were calculated using random-effects models.

**Results:**

Sixteen studies with a total of 41,322 participants were included in the meta-analysis: 10 prospective cohort studies, 5 nested case-control studies and 1 case-control study. Comparing highest vs. lowest category, high S-Cu levels were associated with total stroke (pOR: 1.49, 95% CI 1.22–1.82; *I*^2^ = 0%, *p* = 0.54), myocardial infarction (pOR: 1.31, 95% CI 1.17–1.46; *I*^2^ = 0.0%, *p* = 0.92) and cardiovascular mortality (pOR: 1.60, 95% CI 1.39–1.86; *I*^2^ = 0.0%, *p* = 0.54). Subgroup analysis showed that studies with a hybrid design had higher risks for cardiovascular mortality (pOR: 3.42, 95% CI 1.98–5.92) and ischemic stroke (pOR: 1.54, 95% CI 1.30–1.83).

**Conclusion:**

High S-Cu levels were associated with an increased risk of total stroke, myocardial infarction and cardiovascular mortality. Hybrid studies seems to modify the strength of the association between S-Cu and the risk of cardiovascular mortality and ischemic stroke.

**Systematic review registration:**

[https://www.crd.york.ac.uk/prospero/display_record.php?ID=CRD42022370782], identifier [CRD42022370782].

## Introduction

Cardiovascular disease (CVD) is the leading cause of death and disability worldwide, making it a major public health problem. Despite the existing knowledge about this disease (1, 2), it is expected that around 45% of the American adult population will suffer some type of CVD in 2030 (3, 4). Worldwide, ischemic heart disease and stroke are the first and second causes of death and disability, respectively, in adults older than 50 years (2). It is estimated that around the world there are around 7.74 and 4.19 million incident cases of ischemic and hemorrhagic strokes, respectively. The influence of traditional risk factors on the pathophysiology of CVD has been well documented (5–8). However, the identification of new risk factors is a fundamental aspect in the understanding of new mechanisms associated with the development of CVD. In this sense, the relationship between metals, such as copper, and cardiovascular disease has been described, although the results are not always consistent.

In recent decades, there has been an increase in exposure to certain metal compounds. This has led to a growing interest in learning about the influence of these metals on CVD (9–13). Specifically, copper, despite having a fundamental role in cell metabolism, can also promote oxidation of immediate organic principles through the formation of reactive oxygen species (14–17). The determination of serum copper levels represents the most useful biomarker to know the status of this metal in the body (18).

The relationship between serum copper (S-Cu) levels and CVD has been previously analyzed (19–22). However, this relationship remains uncertain. To date, two meta-analyses (23, 24) have examined the relationship between S-Cu and CVD; however, both studies have shortcomings that limit the quality of the reported evidence, namely: (a) they do not study the association S-Cu and cardiovascular mortality; (b) when studying the S-Cu relationship and stroke, they do not differentiate between ischemic stroke and hemorrhagic stroke; and (c) they ignore potential sources of heterogeneity such as: the epidemiological design of the study, the quality of the study, the S-Cu exposure quantile, the year of publication of the study or the country where it is carried out.

The objectives of this meta-analysis were: (1) To assess the association between S-Cu levels and the risk of major adverse cardiovascular events (MACE), including total stroke, ischemic stroke, hemorrhagic stroke, myocardial infarction, and cardiovascular mortality; and (2) To identify possible sources of heterogeneity for the described association between S-Cu and MACE showed in previous studies.

## Methods

This systematic review and meta-analysis was performed in accordance with the PRISMA statement standards (25). The protocol was previously registered in PROSPERO (CRD42022370782). The focused question was based on Participants/Population, Exposure, Comparator, and Outcome (PECO) strategy: (P) Men and women older than 18 years old; (E) High S-Cu levels; (C) Low S-Cu levels; (O) Total stroke, ischemic or hemorrhagic stroke, myocardial infarction, and cardiovascular mortality.

### Data sources and search strategy

Initially, the search for the scientific literature was carried out through PubMed, Scopus and Web of Science for the period of time between January 1, 1980 and April 20, 2022. The search was kept active from April 21 to May 23, 2023 using the alert systems of each of the electronic platforms. The terms used were: “copper”, “serum copper”, “copper level”, “plasma copper”, “myocardial infarction”, “stroke”, “cardiovascular disease”, and “cardiovascular mortality”. Full information about the search strategy used is shown in Supplementary Table S1. A manual search was also carried out in the references of all the selected articles to ensure that eligible studies were not lost.

### Study selection

The article selection criteria were defined *a priori* as follows: (1) longitudinal observational studies (cohort, case-control and hybrid studies); (2) studies that analyzed the relationship between S-Cu levels (as exposure of interest) and MACE risk (as outcome); (3) by estimating measures of association or to allow its calculation; and (4) studies written in English, French or Spanish. Exclusion criteria were: (a) studies that only looked at the relationship between copper intake and CVD risk; (b) studies that only measured the association of interest in terms of mean difference and standard deviation of copper levels; and (c) letters to the editor, editorials, book chapters, narrative and systematic reviews and trial protocols.

To select the relevant articles, the titles and abstracts were independently reviewed by two investigators (CM-B and ML-L). Duplicate articles were identified and removed. The full text of potentially eligible studies was also assessed by two reviewers (CM-B and ML-L). Disagreements regarding the selection of articles were resolved by a third researcher (JJ-M).

### Data extraction

Two reviewers (CM-B and ML-L) independently extracted data from the included studies using a pre-designed form. The following data were recorded: first author and year of publication, country, study design, quantile of serum copper exposure (tertile, quartile or quintile), sample size, percentage of men, mean age of the participants, serum copper concentration in extreme categories, type of MACE, magnitude for effect measure and 95% CI, and the adjustment variables considered.

### Quality assessment

The quality of the included studies was assessed using the Newcastle-Ottawa scale (26) (NOS) by two investigators (CM-B and ES-I) independently. This scale uses a star system (with a maximum of nine) to assess the quality of a study with respect to three dimensions: (1) selection of study groups, (2) comparability of study groups, and (3) determination of the exposure or outcome of interest for case-control or cohort studies. According to the NOS score, the selected articles were classified into: high (8–9 stars), medium (6–7), or low (5 or less) quality.

### Statistical analysis

The measure of association and 95% confidence interval that estimated the MACE risk were extracted from each of the included studies when comparing the highest S-Cu level category against the lowest (reference category). The association measures of the multivariate models corresponded to those obtained with the model adjusted for the largest number of variables. To obtain the overall pOR, a random effects model was applied and weighted for the variability of the included studies. To assess the heterogeneity of results between the studies, forest plots were examined, and the Cochran Q test was used. *I*^2^ was estimated and heterogeneity was considered as low for values between 25% and 50%, moderate for 50%–75% and high for >75% (27). We explored heterogeneity by stratifying studies based on several potential variables that we assume might have produced the detected heterogeneity. These analyses were conducted considering the following variables: type of study design, sex of participants, country, S-Cu exposure quantile, as well as study quality.

A sensitivity analysis was performed, eliminating those studies that reported abnormally high association measures, to assess the influence of these values on the estimated risk. The possible presence of publication bias was examined by visual inspection of funnel plots. If asymmetry was observed suggestive of a probable publication bias, the Egger test was performed. All statistical tests were two-sided with an *α* level of 0.05. All statistical analyses were conducted using Stata 17.0 (Stata Corp).

## Results

### Identification and selection of studies

**Figure 1:**
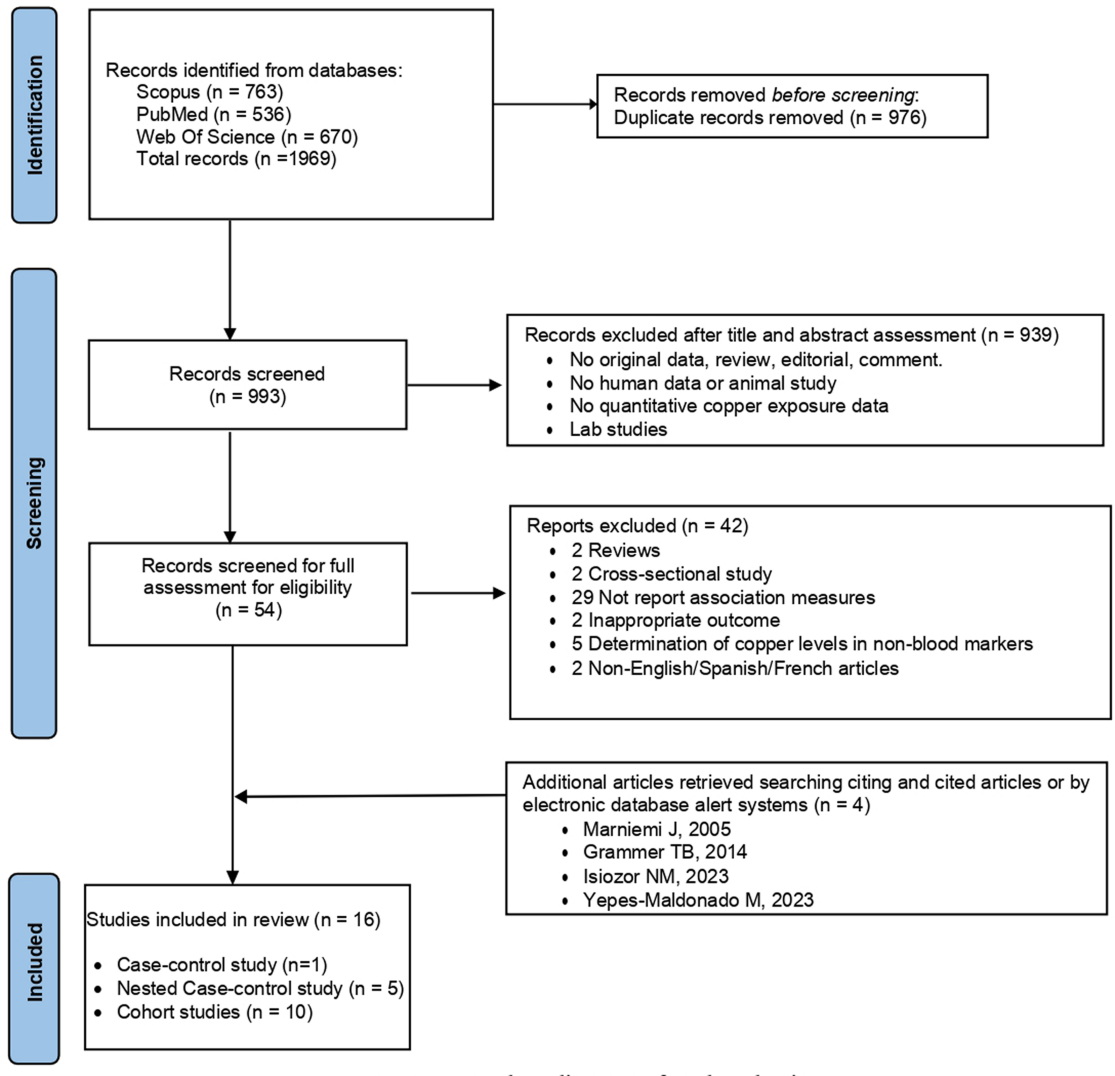
Flow diagram of study selection.

Figure 1 shows a detailed flowchart of the identification and selection of studies. Initially, 1,969 articles were identified as potentially relevant. Of these, 976 articles were eliminated for being duplicates, 939 for not being relevant after reviewing the title and abstract, and 42 after reading the full text. Subsequently, 2 articles found through manual consultation of the included articles' references and 2 through the alert systems activated in the different electronic search platforms used were added. Finally, a total of 16 studies were included in this systematic review and meta-analysis (20–22, 28–39).

### Characteristics of the included studies and quality assessment

The main characteristics of the included studies are shown in Table 1. A total of 41,322 participants were included in the 15 studies. The number of participants in the studies ranged from 186 to 6,155 people, with an age range from 15 to 99 years. The articles were published between 1988 and 2023. Of the total number of studies included, one study evaluated two different cardiovascular events as outcome variables (stroke and myocardial infarction) (21), while the remaining 15 evaluated a single type of cardiovascular event as the outcome variable. Of these, three articles distinguished between total, ischemic and hemorrhagic stroke (35, 37, 40), while one article considered only ischemic stroke (34).

**Table 1:** Characteristics of the studies included in the systematic review and meta-analysis.

First author (year)	Country	Study design	Exposure of S-Cu	Sample size	Male (%)	Mean age of participants (SD)	S-Cu concentration in extreme categories	Type of MACE	Effect estimates (95% CI)	NOS score	Variables adjusted for
Yepes-Calderon M (2023)	Netherlands	Cohort	Continuous	660	56	53 (13)	Median (IQR): 15.42 (13.53–17.63) µmol/L	CM	HR: 1.40 (1.05–1.88)	9	Age, BMI, CVD history, hemoglobin, SBP, sex
Isiozor NM (2023)	Finland	Cohort	Quartile	1,911	100	52.5 (5.3)	Q1 < 1.0; Q4 ≥ 1.21 mg/L	CM	HR: 1.68 (1.23–2.29)	8	Age, alcohol consumption, BMI, CRP, HDL-cholesterol, history of type 2 diabetes, SBP, serum zinc, smoking status, socioeconomic status, total cholesterol.
Shi L (2021)	China	Cohort	Quartile	6,155	50.14	64.90 (7.52)	Q1 < 856.80; Q4 ≥ 1,081.83 µg/L	CM	HR: 1.94 (1.45–2.58)	8	Metals significant in the single-metal model (*p*-trend <0.05) were involved in the Cox proportional hazards model simultaneously (multiple-metals model), stratified by age at risk (in 5-year intervals), sex, and adjusted for BMI, diabetes at baseline, drinking status, education level, eGFR, family history of CVD, future disease status, hyperlipidemia.
Kunutsor SK (2021)	Finland	Cohort	Tertile	1,901	100	Range: 42–61	T1 < 1.03; T3 ≥ 1.18 mg/L	CHD	HR: 1.32 (1.1–1.59)	9	Age, alcohol, BMI, HDL-cholesterol, history of type 2 diabetes, physical activity, SBP, smoking status, total cholesterol.
Hu L (2021)	China	Nested case-control	Quartile	2,510	48.49	Cases: 70.75 (8.07) Controls: 70.76 (8.06)	Q1 < 14.18; Q4 ≥ 17.46 µmol/L	TS	OR: 1.49 (1.16–1.9)	8	Conditioned on the matching factors of age, sex and study site, and adjusted for BMI, drinking status, eGFR, fasting blood glucose, HDL-cholesterol, homocysteine, hypertension, self- reported diabetes, self-reported hyperlipidemia, smoking status, total cholesterol, triglycerides.
								IS	OR: 1.46 (1.12–1.92)		
								HS	OR: 2.05 (0.96–4.38)		
Cabral M (2021)	Germany	Cohort	Quintile	2,464	<1,021 µg/L: 57.9 ≥ 1,021 µg/L: 17.1	<1,021 µg/L: Median (IQR): 49.0 (15.0) ≥1,021 µg/L: Median (IQR): 48.7 (16.1)	Median (IQR): 1,021 (333) µg/L	MI	HR: 1.31 (1.13–1.52)	7	Age, alcohol intake, anti-hypertensive medication, BMI, educational attainment, lipid-lowering medication, mediterranean score, physical activity, prevalent hypertension, sex smoking status, vitamin and mineral preparations, waist circumference.
Xiao Y (2019)	China	Nested case-control	Tertile	2,608	63.1	66.8 (7.5)	IS: T1 < 892.63; T3 > 1,025.91 µg/L	IS	OR: 1.53 (1.2–1.96)	8	BMI, diabetes mellitus, drinking status (current former never), family history of stroke, hyperlipidemia, hypertension, regular exercise, smoking status (current former never).
					58.4	65.62 (7.7)	HS: T1 < 936.36; T3 > 1,073.46 µg/L	HS	OR: 1.06 (0.64–1.74)		
Wen Y (2019)	China	Case-control	Quartile	2,554	57.09	Cases: 59.93 (10.40) Controls: 59.90 (10.24)	Q1 < 751.21; Q4 > 1,029.65 µg/L	IS	OR: 0.99 (0.75–1.29)	8	Alcohol drinking, BMI, diabetes, hyperlipidemia, hypertension, smoking
Zhang J (2019)	China	Nested case-control	Quartile	1,236	47.1	62.3 (7.2)	Q1 < 91.2; Q4 ≥ 117.0 µg/dl	TS	OR: 1.72 (1.12–2.65)	9	Age, alcohol drinking, BMI, eGFR at baseline, fasting glucose, folate, HDL-cholesterol, MTHFR C677T genotypes, SBP, sex, smoking, study site, time-averaged SBP over the treatment period, total cholesterol, total homocysteine, treatment group, triglycerides.
							Q1 < 92.0; Q4 ≥ 117.5 µg/dl	IS	OR: 1.91 (1.18–3.11)		
							Q1 < 86.7; Q4 ≥ 114.1 µg/dl	HS	OR: 1.25 (0.45–3.48)		
Grammer TB (2014)	Germany	Cohort	Quartile	3,253	69.9	Male: 61.8 (10.7) Female: 64.7 (10.2)	Q1 < 91; Q4 ≥ 123 µg/dl	CM	HR: 1.49 (1.10–2.01)	7	Age, BMI, ceruloplasmin, clinical status at presentation (no significant CAD, stable CAD, unstable angina pectoris, STEMI, NSTEMI), CRP, GFR, HDL-cholesterol, hypertension, LDL-cholesterol, sex, smoking status, triglycerides, type 2 diabetes.
Leone N (2006)	France	Cohort	Quartile	4,035	100	Alive: 43 (5) Dead: 44 (4)	Data not show	CM	HR: 1.3 (0.6–2.8)	8	Age, alcohol consumption (≤20 21–40 >40 ml/d), BMI, CVD history (yes/no), diabetes (yes/no), HDL-cholesterol, hypertension (yes/no), LDL-cholesterol, physical activity (sedentary life moderate vigorous exercise), smoking status (never former or current), triglycerides.
Marniemi J (2005)	Finland	Cohort	Tertile	755	47.81	Range: 65–99	Data not show	TS	HR: 1.15 (0.65–2.03)	6	Age, functional capacity, sex, smoking.
								MI	HR: 1.2 (0.77–1.85)		
Ford ES (2000)	U.S.A	Cohort	Quartile	4,574	Alive: 46.2 Dead: 74.1	Alive: mean(se): 47.2 (0.3) Dead: mean(se): 62.2 (1)	Q1 < 106; Q4 ≥ 137 µg/dl	CM	HR: 2.87 (1.57–5.25)	8	Age, BMI, education, HDL-cholesterol, history of diabetes, nonrecreational activity, race, recreational activity, SBP, sex, smoking status, total cholesterol, white blood cell count.
Reunanen A (1996)	Finland	Nested case-control	Tertile	504	100	Range: 15–69	T1 < 16.2; T3 > 19.4 µmol/L	CM	OR: 3.38 (1.7–6.7)	6	Age, BMI, heart disease, hypertension, smoking, social class, total cholesterol.
Salonen JT (1991)	Finland	Cohort	Tertile	1,666	100	52.3 (5)	T1 < 1.02; T3 ≥ 1.16 mg/L	MI	HR: 4 (1.5–10.8)	8	Age, blood leukocyte, blood leukocyte count, cigarette, diabetes, examination year, family history of ischemic heart disease, HDL-cholesterol, ischemic electrocardiogram in exercise, LDL-cholesterol, maximal oxygen uptake, SBP.
Kok FJ (1988)	Netherlands	Nested case- control	Quintile	186	53.2	Case: 68.2 (12.1) Controls: 67.8 (11.8)	Q1 < 1.05; Q5 > 1.43 mg/L	CM	OR: 3.5 (1.4–8.7)	8	Antioxidants (selenium, vitamin A and vitamin E), BMI, DBP, SBP, smoking, total cholesterol, week of blood collection, years of education, zinc.

S-Cu indicates serum copper; MACE, major adverse cardiovascular event; NOS, New Castle-Ottawa Scale; CM, cardiovascular mortality; CHD, cardiovascular heart disease; TS, total stroke; IS, ischemic stroke; HS, hemorrhagic stroke; MI, myocardial infarction; HR, hazard ratio; OR, odds ratio; BMI, body mass index; CRP, C-reactive protein; HDL, high-density lipoprotein; SBP, systolic blood pressure; eFGR, estimated glomerular filtration rate; CVD, cardiovascular disease; MTHFR, methylenetetrahydrofolate reductase; CAD, coronary artery disease; STEMI, ST-segment elevation myocardial infarction; NSTEMI, non-ST-segment elevation myocardial infarction; FGR, glomerular filtration rate; LDL, low-density lipoprotein; DBP, diastolic blood pressure.

On the other hand, eight studies evaluated cardiovascular mortality as the main outcome (20, 22, 28, 30–32, 39) two the risk of myocardial infarction (29, 36), and one the risk of coronary artery disease (38). Regarding the geographical location, five studies were conducted in the Asian population (China) (22, 34, 35, 37, 40), ten in the European population [Finland (*n *= 5) (21, 29, 30, 38, 39), Germany (*n *= 2) (32, 36), France (*n *= 1) (31) and 1 Netherlands (*n *= 2) (28, 41)] and one in the American population (USA) (20). Regarding the sex of the participants, five studies included only men (29–31, 38, 39). Regarding the design, ten were from cohort studies (20–22, 29, 31, 32, 36, 38, 39) five nested case-control (28, 30, 35, 37, 40), and one case-control (34). Twelve of the fifteen articles included (75%) were assessed as high quality, the rest (25%) as medium quality (Supplementary Table S2).

### Risk of stroke

**Figure 2:**
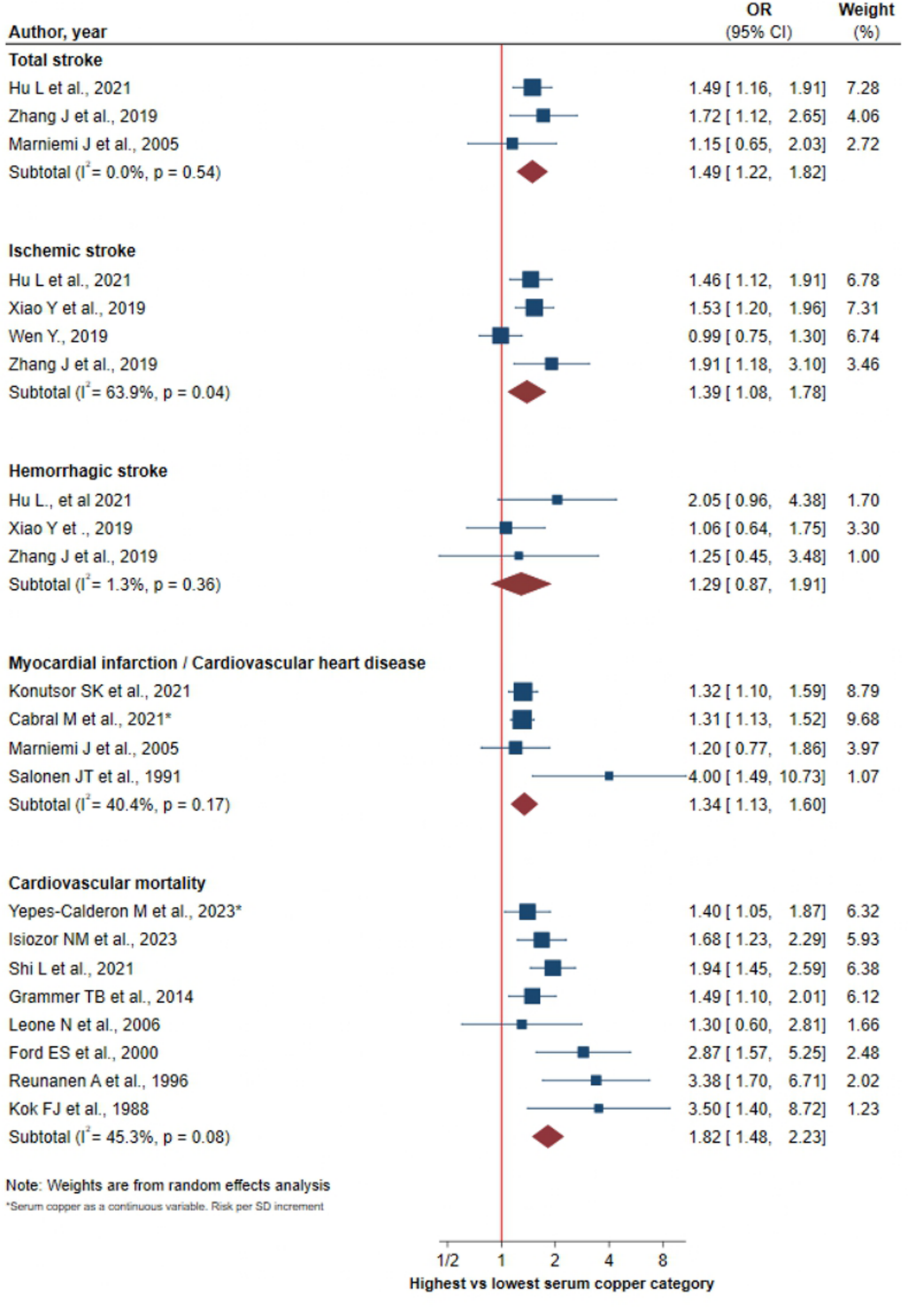
Forest plot of the association between serum copper levels and MACE.

The overall pOR of the association between S-Cu and the risk of total stroke, ischemic stroke, and hemorrhagic stroke was 1.49 (95% CI 1.22–1.82), 1.39 (95% CI 1.08–1.78) and 1.29 (95% CI 0.87–1.91), respectively (Figure 2). Significant heterogeneity was observed between studies for ischemic stroke (*I*^2^ = 69.3%, *p* = 0.04), with no heterogeneity being found for total stroke and hemorrhagic stroke. Visual inspection of funnel plots and Egger test revealed no evidence of publication bias (Supplementary Figures S1–S3).

### Risk of myocardial infarction

A significant association was found between S-Cu levels and the risk of heart attack from myocardium/coronary disease (pOR: 1.34, 95% CI 1.13–1.60) with a medium-low heterogeneity among studies (*I*^2^ = 40.4%, *p* = 0.17) (Figure 2). Both the funnel plot and the Egger test (*p* = 0.1203) did not clearly show the existence of publication bias. Although the “abnormal” value belonging to the study by Salonen et al. (29) clearly impacted with the *p* value of the Egger's test not being superior (Supplementary Figures S4, S5).

### Risk of cardiovascular mortality

S-Cu levels were positively associated with cardiovascular mortality risk (pOR: 1.82, 95% CI 1.48–2.23), showing medium-low heterogeneity among studies (*I*^2^ = 45.3%, *p* = 0.08) (Figure 2). The funnel plot showed a slight asymmetry corroborated by Egger's test (*p* = 0.039) (Supplementary Figure S6).

### Sensitivity analysis and subgroup analysis

**Figure 3:**
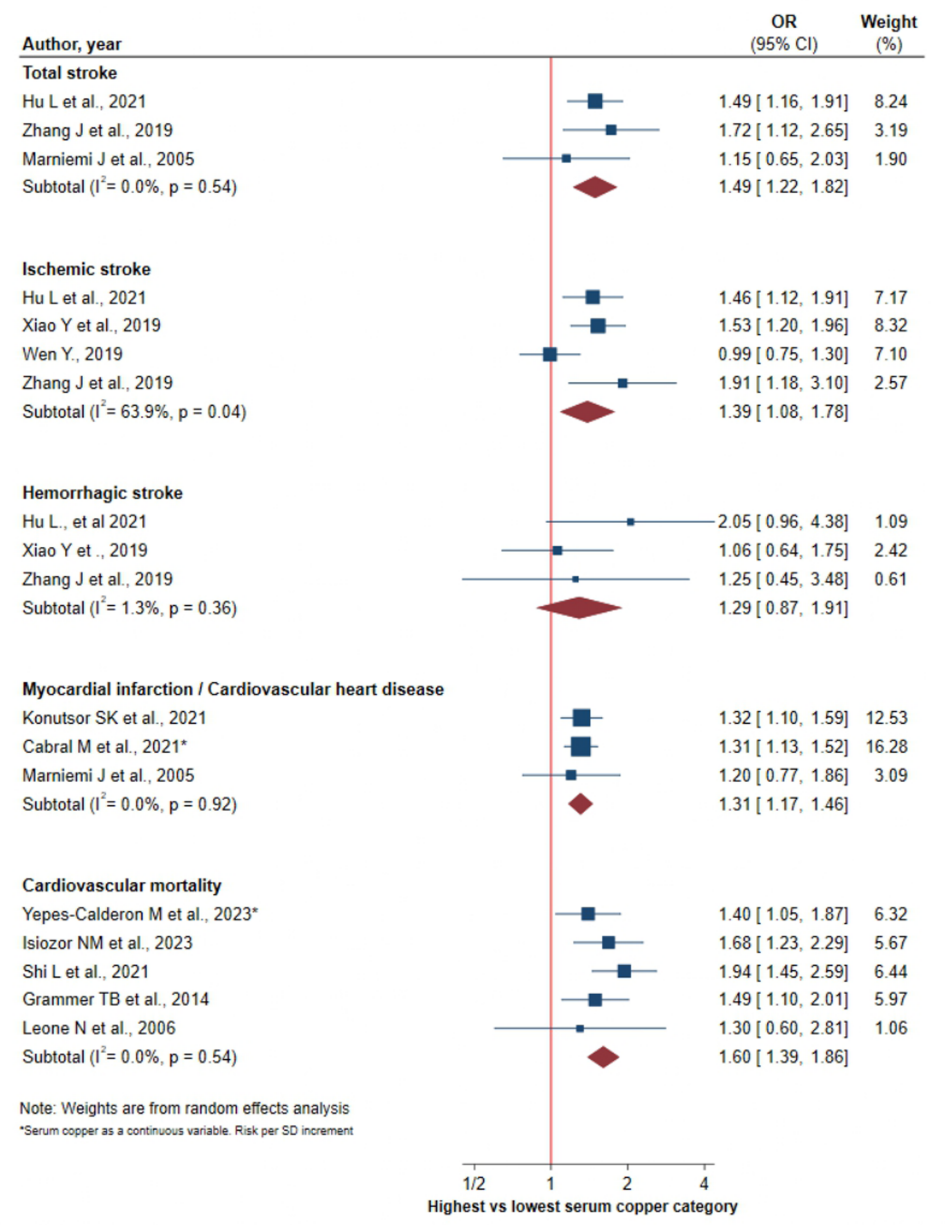
Forest plot of the association between serum copper levels and MACE without the studies before 2005.

Of the sixteen studies included in the meta-analysis, the four studies published before from 2005 (20, 28–30) showed a particularly high magnitude of the effect measure, OR equal or higher than 2.87. Sensitivity analysis revealed a decreased risk of cardiovascular mortality, 1.60 (95% CI 1.39–1.86) vs. 1.82 for the total of the studies although the confidence intervals are overlapped. On the other hand, the risk of myocardial infarction was virtually unchanged, 1.31 (95% CI 1.17–1.46) (Figure 3). The previously observed heterogeneity for cardiovascular mortality risk was eliminated following this sensitivity analysis, as well as the disappearance of publication bias (Supplementary Figure S7).

Table 2 presents the subgroup analysis for cardiovascular mortality. The direct association observed between S-Cu and cardiovascular mortality is confirmed in most of the subgroups established according to the participants' sex, country, and S-Cu exposure quantile. However, in the analysis by study design, the risk of cardiovascular mortality was significantly higher in studies with a hybrid design compared to cohort studies (pOR: 3.42, 95% CI 1.98–5.92; *I*^2^ = 0%, *p* = 0.95 vs. pOR: 1.67, 95% CI 1.41–1.97; *I*^2^ = 22.9%, *p* = 0.26). In the analysis based on the quality of the studies, the association was significant for high quality studies (pOR: 1.80, 95% CI 1.44–2.25; *I*^2^ = 38.9%, *p* = 0.15), but not for medium quality (pOR: 2.11, 95% CI 0.96–4.67; *I*^2^ = 78.2%, *p* = 0.03). A greater strength of the association, relative to the overall pooled effect, was also observed in the studies conducted in Finland. For ischemic stroke, the subgroup analysis revealed a slightly increased risk in studies with a hybrid design compared with case-control studies (pOR: 1.54, 95% CI 1.30–1.83; *I*^2^ = 0%, *p* = 0.63 vs. pOR: 0.99, 95% CI 0.75–1.30) (Table 3). Regarding myocardial infarction, no source of heterogeneity was identified (Supplementary Table S3).

**Table 2:** Subgroup analysis of risk of cardiovascular mortality.

	No of studies	OR (95% CI)	*p*-value	Heterogeneity
Epidemiologic study design				
Case-control	0	-	-	-
Nested case-control	2	3.42 (1.98–5.92)	<0.001	*I*^2 ^= 0%
Prospective cohort	6	1.67 (1.41–1.97)	<0.001	*I*^2^ = 22.9%
Sex				
Female	0	-	-	-
Male	3	1.91 (1.19–3.06)	0.007	*I*^2^ = 51.4%
Both	5	1.81 (1.40–2.34)	<0.001	*I*^2^ = 53.2%
Country				
China	1	1.94 (1.45–2.59)	<0.001	-
Finland	2	2.22 (1.14–4.35)	0.020	*I*^2^ = 69.8%
France	1	1.30 (0.60–2.81)	0.504	-
USA	1	2.87 (1.57–5.25)	0.001	-
Netherlands	2	1.99 (0.83–4.77)	0.123	*I*^2^ = 71.5%
Germany	1	1.49 (1.10–2.01)	0.010	-
Quantile of serum copper				
Tertile	1	3.38 (1.70–6.71)	<0.001	-
Quartile	5	1.75 (1.46–2.11)	<0.001	*I*^2^ = 16.7%
Quintile	1	3.50 (1.40–8.72)	0.007	-
Continuous	1	1.40 (1.05–1.87)	0.024	-
NOS scale category				
Low	0	-	-	-
Moderate	2	2.11 (0.96–4.67)	0.065	*I*^2^ = 78.2%
High	6	1.80 (1.44–2.25)	<0.001	*I*^2^ = 38.9%

**Table 3:** Subgroup analysis of risk of ischemic stroke.

	No of studies	OR (95% CI)	*p*-value	Heterogeneity
Epidemiologic study design				
Case-control	1	0.99 (0.75–1.30)	0.942	-
Nested case-control	3	1.54 (1.30–1.83)	<0.001	*I*^2^ = 0%
Prospective cohort	0	-	-	-
Sex				
Female	0	-	-	-
Male	0	-	-	-
Both	4	1.39 (1.08–1.78)	0.010	*I*^2^ = 63.9%
Country				
China	4	1.39 (1.08–1.78)	0.010	*I*^2^ = 63.9%
Finland	0	-	-	-
France	0	-	-	-
USA	0	-	-	-
Netherlands	0	-	-	-
Germany	0	-	-	-
Quantile of serum copper				
Tertile	1	1.53 (1.20–1.96)	0.001	-
Quartile	3	1.35 (0.95–1.92)	0.095	*I*^2^ = 71.4%
Quintile	0	-	-	-
NOS scale category				
Low	0	-	-	-
Moderate	0	-	-	-
High	4	1.39 (1.08–1.78)	0.010	*I*^2^ = 63.9%

## Discussion

To the best of our knowledge, this is the first meta-analysis that, in the context of studying the relationship between S-Cu and MACE, examines the relationship between S-Cu and cardiovascular mortality, as well as between S-Cu and stroke, distinguishing between ischemic stroke and hemorrhagic stroke. Within population reference values, a direct association was observed between S-Cu levels and the risk of total stroke, myocardial infarction and cardiovascular mortality. No association was found between S-Cu and hemorrhagic stroke or ischemic stroke. The observed heterogeneity for myocardial infarction and cardiovascular mortality disappeared after the influence analysis was performed in which studies reporting particularly high measures of association were removed. Finally, based on the subgroup analysis, our findings showed that studies with a hybrid design described higher risks for cardiovascular mortality and ischemic stroke.

Observational studies have previously analyzed the relationship between copper levels and CVD, obtaining contradictory results. This is observed in those studies where exposure to copper has been determined using serum concentration. Several of these studies suggest an increased risk of CVD associated with high levels of S-Cu (20, 22, 32, 38, 39), while other researchers have not found any such association (19, 21, 31, 34). Likewise, contradictory results have been reported in studies that analyzed the relationship between dietary consumption of copper and CVD (42–47).

Copper is an essential element for the organism that forms part of proteins with important biological functions, such as ceruloplasmin, superoxide dismutase, cytochrome c oxidase or lysyl oxidase (48). However, under certain circumstances it can also be harmful to the body, as it can favor the formation of reactive oxygen species through Fenton-type redox reactions (49, 50). It is through this mechanism that copper contributes to the development of the atherosclerotic process, which lays the foundation for cardiovascular disease (51–53). Other studies have indicated the involvement of copper in the pathophysiology of CVD, through the formation of the copper-homocysteine complex, favoring endothelial dysfunction (54, 55). Likewise, a positive association has been observed between S-Cu levels and acute phase proteins during the inflammatory process in the context of CVD (20, 31, 32, 56). Other investigations have reported an association between copper levels and an increased prevalence of certain cardiovascular risk factors such as hyperlipidemia (37, 52), hypertension or high fasting blood glucose, among others (37). High levels of S-Cu have also been related to patients with type 2 diabetes mellitus (43). In patients with Wilson's disease, characterised by excessively high copper levels due to a genetic disorder, cardiac disorders such as mild left ventricular hypertrophy, benign supraventricular tachycardias and extrasystolic beats have been reported, providing evidence that copper may be a risk factor for cardiovascular disease (57, 58).

Two previous meta-analyses have examined the relationship of copper levels on cardiovascular events, although they have limitations that should be considered. Bao's meta-analysis (23) focuses on analysing the relationship between copper levels and stroke, but does not differentiate between hemorrhagic and ischemic events. For its part, the Chowdhury's meta-analysis (24) gives estimates for which it does not assess the presence of heterogeneity, for example a pOR of 2.22 is given for coronary heart disease with an *I*^2^ of 66.7% and a *p* for heterogeneity of 0.3. Moreover, in the latter meta-analysis, stroke risk and stroke mortality are considered as if they were the same event. Limitations that cast doubt on the estimates made by both reviews on the relationship between copper and the different cardiovascular events.

We decided not to conduct a dose-response meta-analysis for the following reasons: (1) Not all studies classify serum copper levels in the same way. There are studies that work with tertiles, others with quartiles, one with quintiles. And there are even two studies that work with the variable continuously; (2) For those studies that use the same way of classifying blood copper exposure levels, the cut-off points are different and therefore we consider that they should not be used to analyse a dose-response gradient; (3) Moreover, some studies do not report the cut-off points for the different quantiles; (4) Not all studies give the median of serum copper for the different categories; and (5) To estimate a dose-response meta-analysis is convenient to know the number of cases by category. In this sense, some articles do not show the number of cases per quantile.

This study presents some limitations that must be considered in the interpretation of the results. First, our review was based solely on data from observational studies, an aspect that must be taken into account when assessing the level of causality. Second, although most of the articles estimated measures of association using multivariable regression models, the presence of residual confounding cannot be ruled out. In this sense, the lack of adjustment for other metals, such as zinc or other variables such as dietary pattern or total energy intake, could affect the independence of the association between S-Cu and MACE risk. Third, the restriction in the language may have limited the number of studies included in the meta-analysis; however, the number of articles published in languages other than those considered in this study is most likely very low.

The present meta-analysis also has several strengths. First, to the best of our knowledge, it is the first meta-analysis that, in the context of the study of the relationship between S-Cu and MACE, examines the relationship between S-Cu and cardiovascular mortality and between S-Cu and stroke, distinguishing between ischemic stroke and hemorrhagic stroke. Second, the type of study design has been identified as a source of heterogeneity, which has not been described to date, for cardiovascular mortality and ischemic stroke. Third, to control for confounding bias, the measures of association considered have been those from the maximum fit models. Finally, and to the best of our knowledge, these data provide the most complete information to date on the association between S-Cu and MACE risk.

## Conclusion

We found a positive association between S-Cu levels and the risk of total stroke, myocardial infarction and cardiovascular mortality. No association was found for hemorrhagic stroke or ischemic stroke. The epidemiological design of the studies seems to modify the strength of the association between S-Cu and the risk of cardiovascular mortality and ischemic stroke. Monitoring of S-Cu levels, as an independent risk marker, could be an additional tool in the primary prevention of MACE.

## Data Availability

The original contributions presented in the study are included in the article/**Supplementary Material**, further inquiries can be directed to the corresponding author.
