# Supplementary material for: Serum copper levels and risk of major adverse cardiovascular events: a systematic review and meta-analysis
Source: Front Cardiovasc Med. 2023 Jun 27;10:1217748. doi: 10.3389/fcvm.2023.1217748 (PMC10333529; doi:10.3389/fcvm.2023.1217748)
Supplement: Supplementary file 1 [file Table1.docx]

**TABLE S1:** Searching strategy^*^: Medical Subject Headings (MeSH) and non-MeSH keywords used to search relevant publications

| Concept 1 | ("copper"[MeSH Terms] OR "serum copper"[Title/Abstract] OR "copper level"[Title/Abstract] OR "plasma copper"[Title/Abstract] OR "copper"[Title/Abstract]) |
| --- | --- |
| Concept 2 | ("myocardial infarction"[MeSH Terms] OR "stroke"[MeSH Terms] OR "myocardial infarction"[Title/Abstract] OR "stroke"[Title/Abstract] OR "cardiovascular disease"[Title/Abstract] OR "cardiovascular mortality"[Title/Abstract]) |
| Used strategy | (Concept 1 AND concept 2) |

^*^The combination of keywords as mentioned above was used to search online databases
