# Supplementary material for: Serum copper levels and risk of major adverse cardiovascular events: a systematic review and meta-analysis
Source: Front Cardiovasc Med. 2023 Jun 27;10:1217748. doi: 10.3389/fcvm.2023.1217748 (PMC10333529; doi:10.3389/fcvm.2023.1217748)
Supplement: Supplementary file 2 [file Table2.docx]

**TABLE S2**. Quality assessment of the studies included in the systematic review and meta-analysis

| Cohort studies | | | | | | | | | | |
| --- | --- | --- | --- | --- | --- | --- | --- | --- | --- | --- |
|  | **Selection** | | | | **Comparability** | | **Outcome** | | | **TS*** |
| Study (year) | Representativeness of exposed cohort | Representativeness of unexposed cohort | Ascertainment of exposure | Outcome was not present at start | Important Factor | Additional Factor | Assessment | Exposure follow-up long enough for outcomes to occur | Adequacy of follow-up |  |
| Yepes-Maldonado M (2023) | 🟊 | 🟊 | 🟊 | 🟊 | 🟊 | 🟊 | 🟊 | 🟊 | 🟊 | 9 |
| Isiozor NM (2023) |  | 🟊 | 🟊 | 🟊 | 🟊 | 🟊 | 🟊 | 🟊 | 🟊 | 8 |
| Shi L (2021) |  | 🟊 | 🟊 | 🟊 | 🟊 | 🟊 | 🟊 | 🟊 | 🟊 | 8 |
| Kunutsor SK (2021) | 🟊 | 🟊 | 🟊 | 🟊 | 🟊 | 🟊 | 🟊 | 🟊 | 🟊 | 9 |
| Cabral M (2021) | 🟊 | 🟊 | 🟊 |  | 🟊 | 🟊 | 🟊 |  | 🟊 | 7 |
| Grammer TB (2014) |  |  | 🟊 | 🟊 | 🟊 | 🟊 | 🟊 | 🟊 | 🟊 | 7 |
| Leone N (2006) | 🟊 |  | 🟊 | 🟊 | 🟊 | 🟊 | 🟊 | 🟊 | 🟊 | 8 |
| Marniemi J (2005) |  | 🟊 | 🟊 |  |  | 🟊 | 🟊 | 🟊 | 🟊 | 6 |
| Ford ES (2000) | 🟊 | 🟊 | 🟊 | 🟊 | 🟊 | 🟊 | 🟊 |  | 🟊 | 8 |
| Salonen JT (1991) | 🟊 | 🟊 | 🟊 | 🟊 | 🟊 | 🟊 | 🟊 |  | 🟊 | 8 |
|  | | | | | | | | | | |
| Case-control studies | | | | | | | | | | |
|  | **Selection** | | | | **Comparability** | | **Exposure** | | | **TS*** |
| Study (year) | Definition of cases | Representativeness of cases | Selection of controls | Definition of controls | Important Factor | Additional Factor | Ascertainment | Same methos for subjects | Non-response date |  |
| Hu L (2021) | 🟊 | 🟊 |  | 🟊 | 🟊 | 🟊 | 🟊 | 🟊 | 🟊 | 8 |
| Xiao Y (2019) | 🟊 | 🟊 |  | 🟊 | 🟊 | 🟊 | 🟊 | 🟊 | 🟊 | 8 |
| Wen Y (2019) | 🟊 | 🟊 |  | 🟊 | 🟊 | 🟊 | 🟊 | 🟊 | 🟊 | 8 |
| Zhang J (2019) | 🟊 | 🟊 | 🟊 | 🟊 | 🟊 | 🟊 | 🟊 | 🟊 | 🟊 | 9 |
| Reunanen A (1996) | 🟊 |  |  |  | 🟊 | 🟊 | 🟊 | 🟊 | 🟊 | 6 |
| Kok FJ (1988) | 🟊 |  | 🟊 | 🟊 | 🟊 | 🟊 | 🟊 | 🟊 | 🟊 | 8 |

* Total score
