# Supplementary material for: Serum copper levels and risk of major adverse cardiovascular events: a systematic review and meta-analysis
Source: Front Cardiovasc Med. 2023 Jun 27;10:1217748. doi: 10.3389/fcvm.2023.1217748 (PMC10333529; doi:10.3389/fcvm.2023.1217748)
Supplement: Supplementary file 3 [file Table3.docx]

**TABLE S3**. Subgroup analysis of risk of myocardial infarction.

|  | **Nº of studies** | **OR (95% CI)** | **p-value** | **Heterogeneity** |
| --- | --- | --- | --- | --- |
| **Epidemiologic study design** |  |  |  |  |
| Case-control | 0 | - | - | - |
| Nested case-control | 0 | - | - | - |
| Prospective cohort | 4 | 1.34 (1.13-1.60) | 0.001 | I^2^ = 40.4% |
| **Sex** |  |  |  |  |
| Female | 0 | - | - | - |
| Male | 2 | 2.06 (0.71-5.97) | 0.184 | I^2^ = 78.7% |
| Both | 2 | 1.30 (1.13-1.49) | <0.001 | I^2^ = 0% |
| **Country** |  |  |  |  |
| China | 0 | - | - | - |
| Finland | 3 | 1.48 (0.98-2.22) | 0.060 | I^2^ = 59.9% |
| France | 0 | - | - | - |
| USA | 0 | - | - | - |
| Netherlands | 0 | - | - | - |
| Germany | 1 | 1.31 (1.13-1.52) | <0.001 | - |
| **Quantile of serum copper** |  |  |  |  |
| Tertile | 3 | 1.48 (0.98-2.22) | 0.060 | I^2^ = 59.9% |
| Quartile | 0 | - | - | - |
| Quintile | 1 | 1.31 (1.13-1.52) | <0.001 | - |
| **NOS scale category** |  |  |  |  |
| Low | 0 | - | - | - |
| Moderate | 2 | 1.30 (1.13-1.49) | <0.001 | I^2^ = 0% |
| High | 2 | 2.06 (0.71-5.97) | 0.184 | I^2^ = 78.7% |
