# Supplementary material for: Serum copper levels and risk of major adverse cardiovascular events: a systematic review and meta-analysis
Source: Front Cardiovasc Med. 2023 Jun 27;10:1217748. doi: 10.3389/fcvm.2023.1217748 (PMC10333529; doi:10.3389/fcvm.2023.1217748)
Supplement: Supplementary file 4 [file Datasheet1.docx]

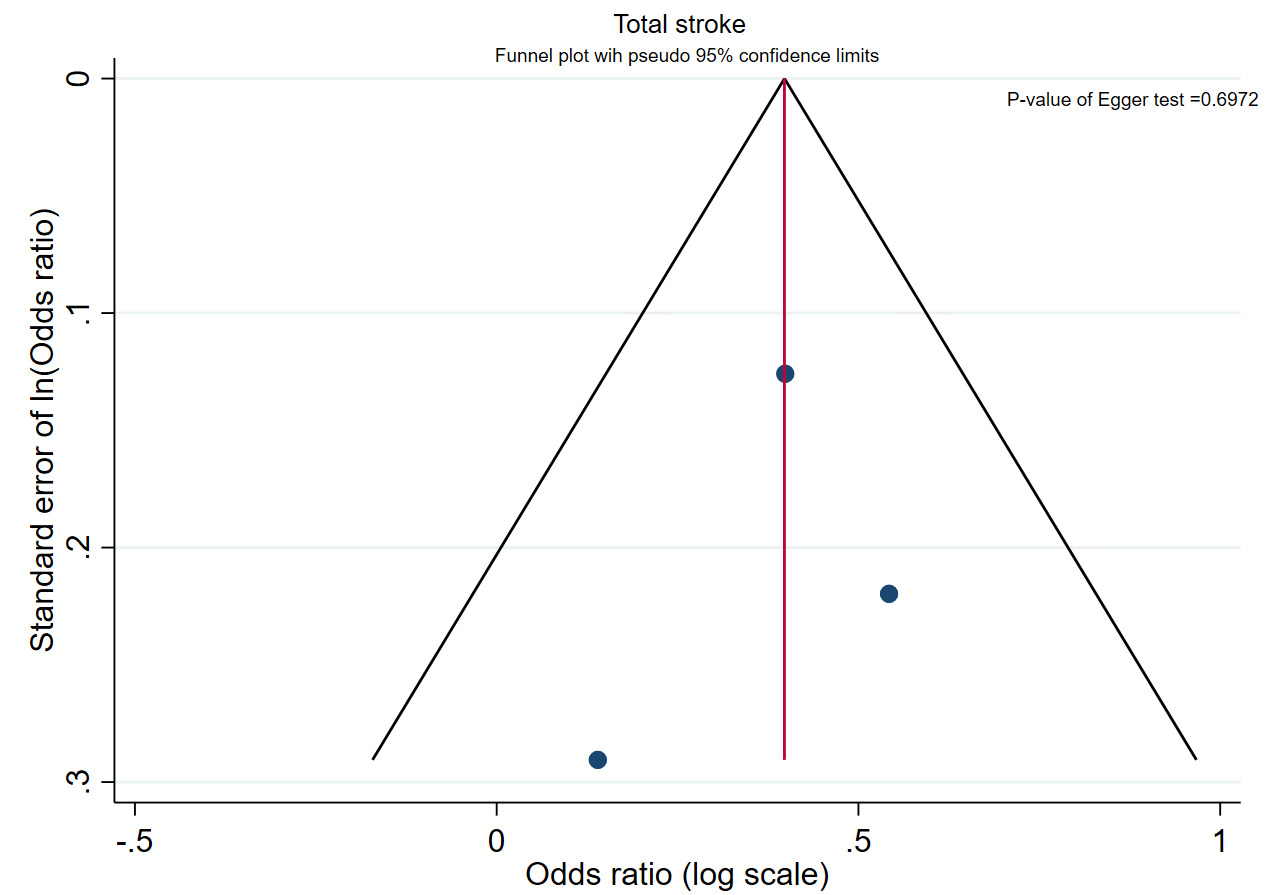


**FIGURE S1.** Funnel plot for assessing publication bias of studies that analyzes the relationship between serum copper and total stroke.


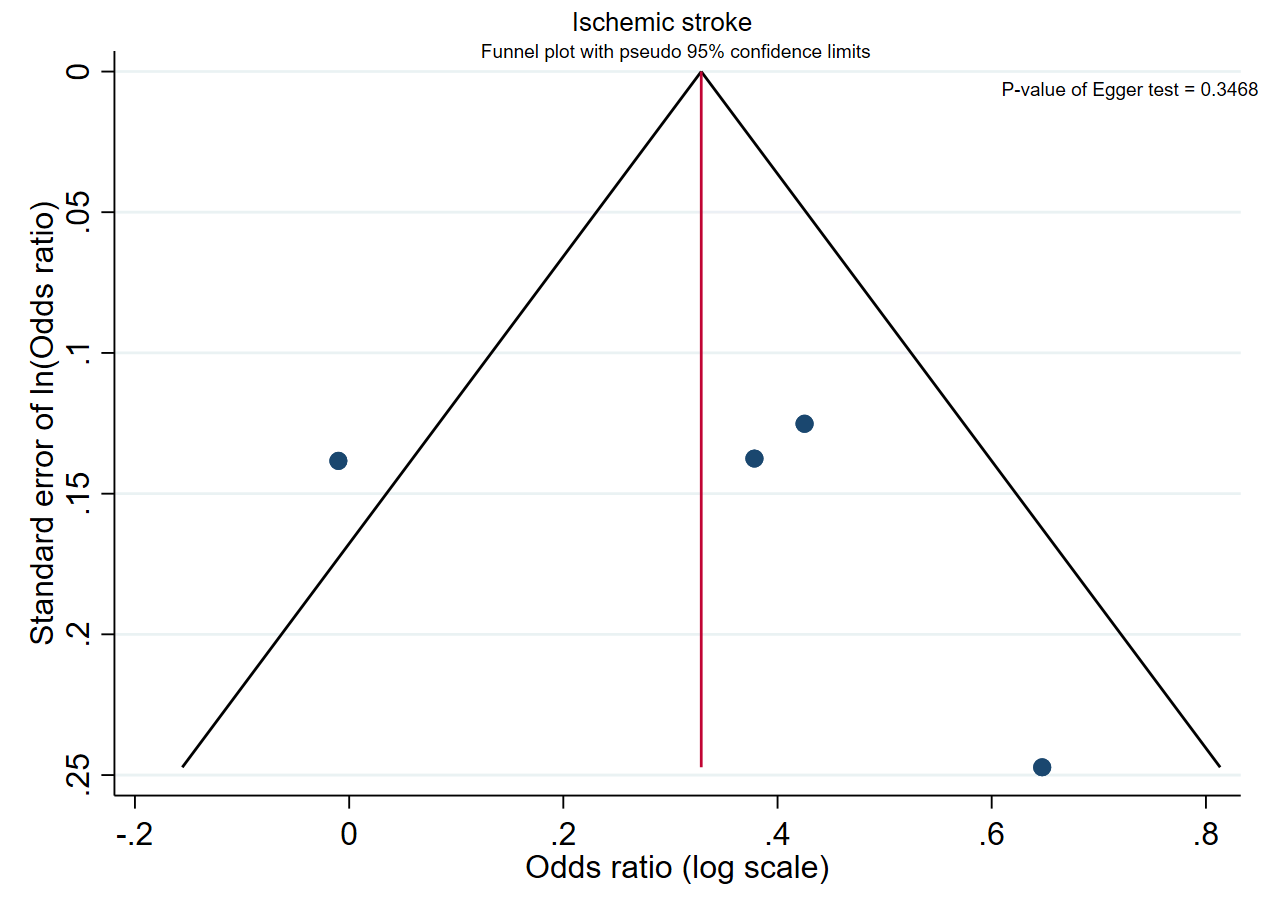
**FIGURE S2.** Funnel plot for assessing publication bias of studies that analyzes the relationship between serum copper and ischemic stroke.


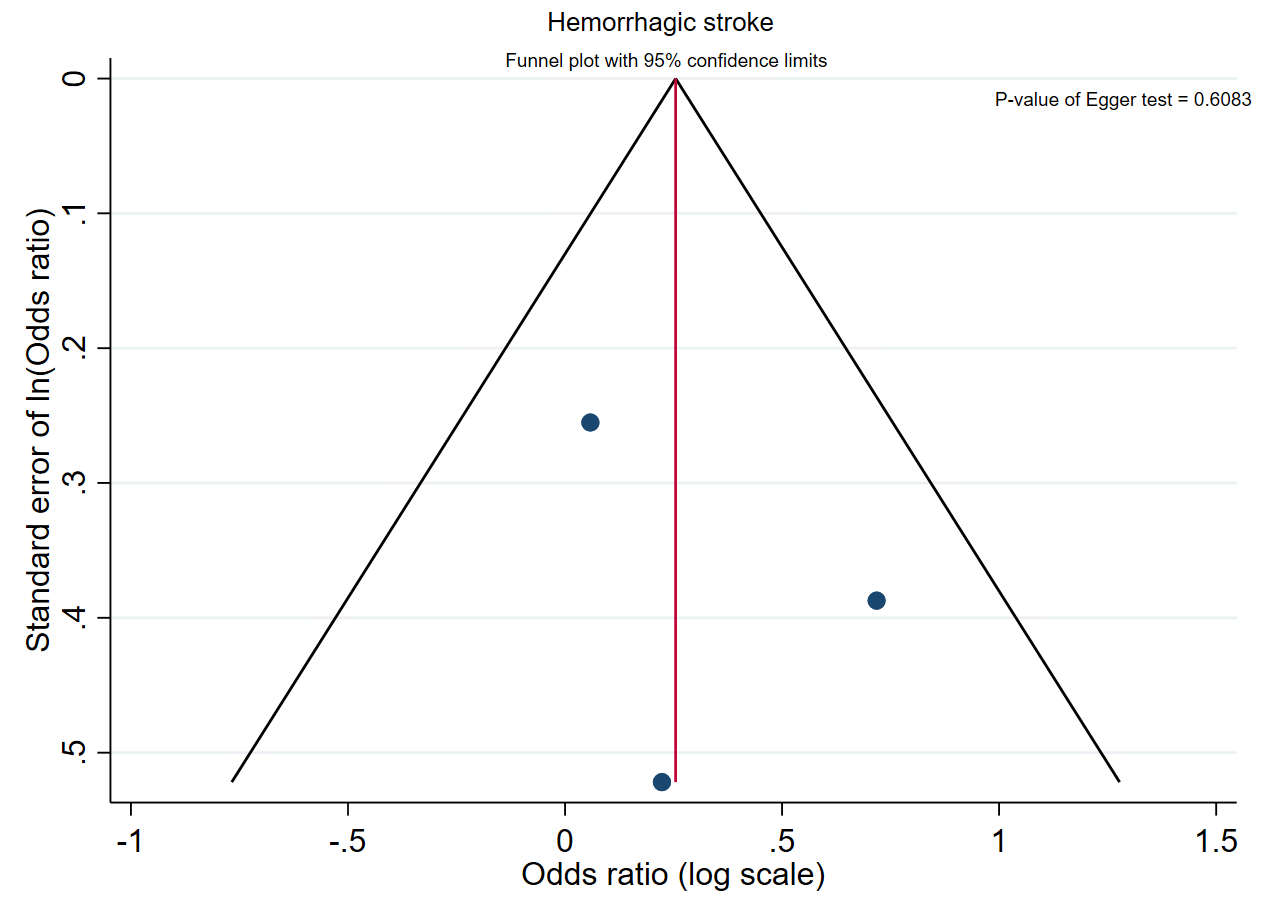
**FIGURE S3.** Funnel plot for assessing publication bias of studies that analyzes the relationship between serum copper and hemorrhagic stroke.
