# Supplementary material for: Serum copper levels and risk of major adverse cardiovascular events: a systematic review and meta-analysis
Source: Front Cardiovasc Med. 2023 Jun 27;10:1217748. doi: 10.3389/fcvm.2023.1217748 (PMC10333529; doi:10.3389/fcvm.2023.1217748)
Supplement: Supplementary file 6 [file Datasheet3.docx]

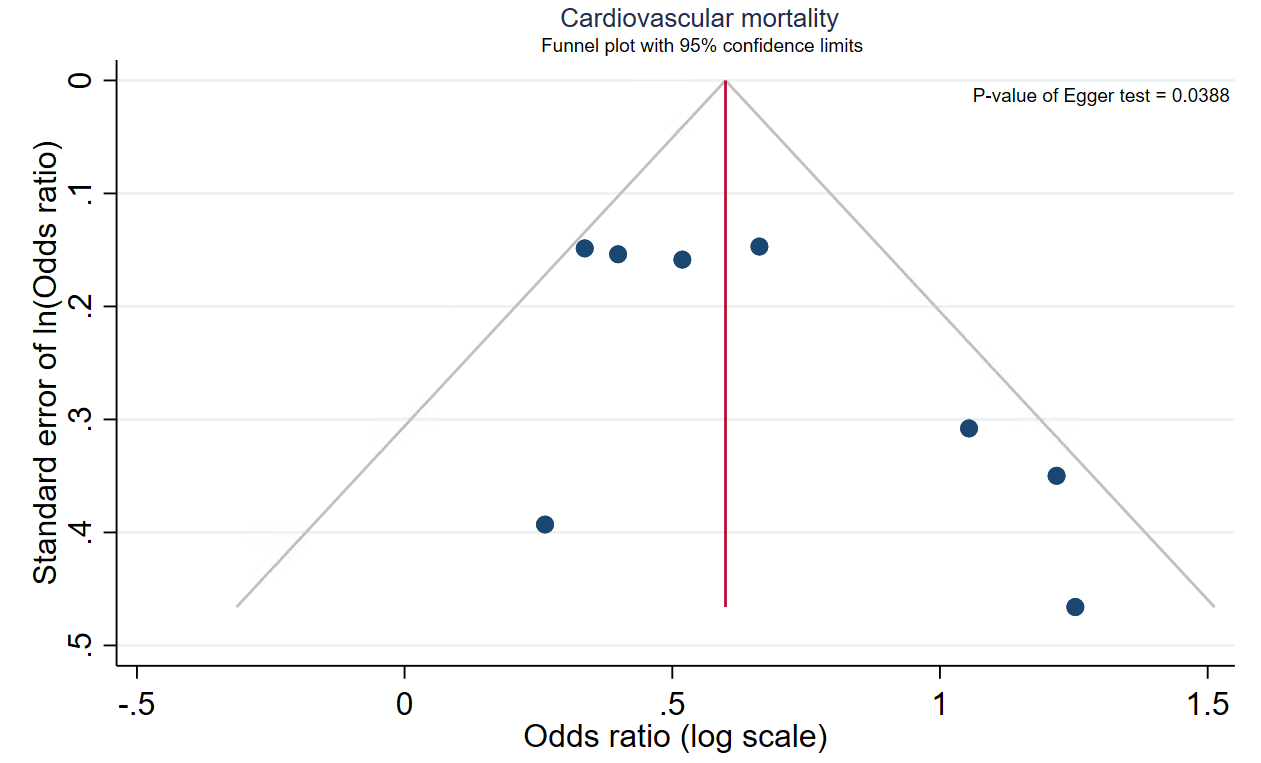
**FIGURE S6.** Funnel plot for assessing publication bias of studies that analyzes the relationship between serum copper and cardiovascular mortality.


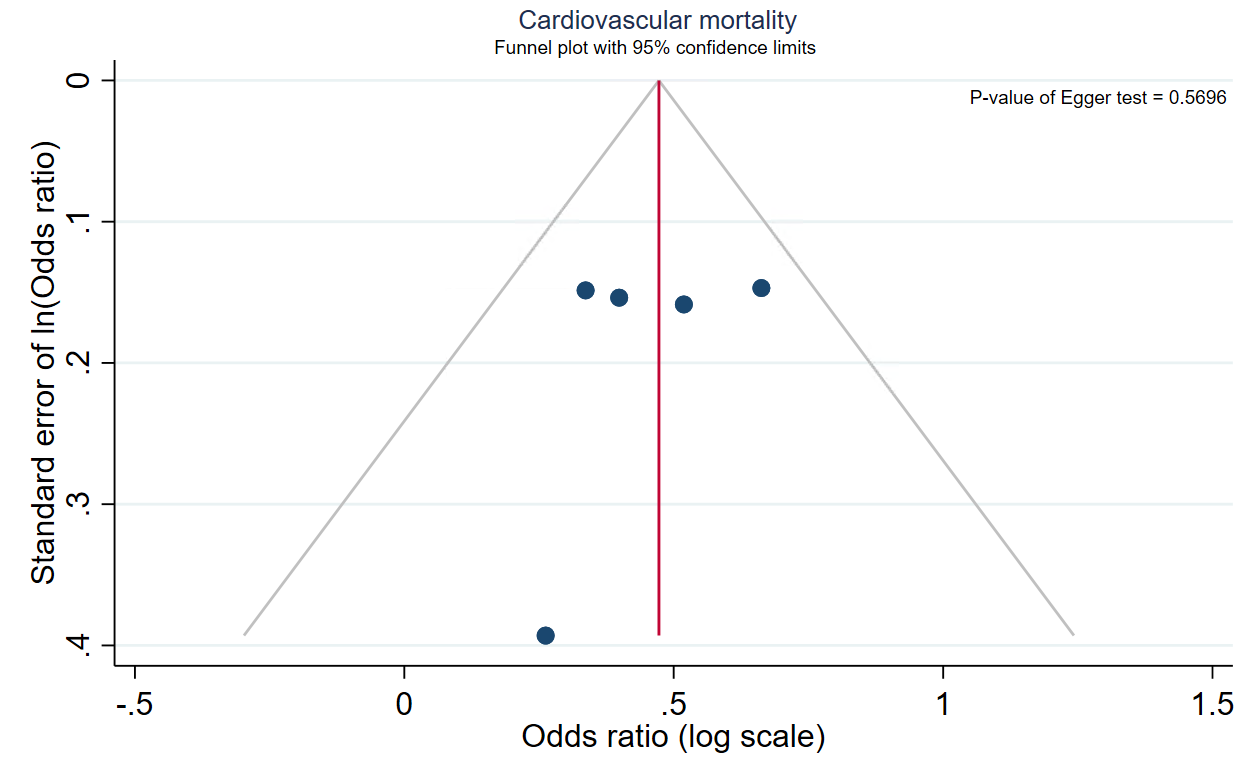
**FIGURE S7.** Funnel plot for assessing publication bias of studies that analyzes the relationship between serum copper and cardiovascular mortality without the studies published before 2005.
